# Spatial structure evolution and ecosystem service relationship changes in urban-fringe-rural areas of megacities: Evidence from Suzhou, China

**DOI:** 10.1371/journal.pone.0332934

**Published:** 2025-09-24

**Authors:** Qianhong Mao, Yasi Tian

**Affiliations:** 1 Department of Urban and Rural Planning, School of Architecture, Soochow University, Suzhou, CHINA; 2 Center for Chinese Urbanization Studies, Soochow University, Suzhou, CHINA; Wuhan University, CHINA

## Abstract

The evolution of urban-fringe-rural structures profoundly impacts ecosystem services (ESs). However, the way in which trade-offs and synergies in ESs respond to changes in regional spatial structures has rarely been discussed. This knowledge gap hinders the development of spatially explicit strategies to mitigate ecological degradation while accommodating urban growth, ultimately perpetuating unsustainable landscape management practices characterized by reactive rather than preventive interventions. Such critical disconnect between structural dynamics and ES feedbacks has emerged as a major bottleneck to operationalizing landscape sustainability in metropolitan regions. This study selected Suzhou—a typical megacity in China—as an example to conduct an empirical study. The urban, urban fringe, and rural areas were firstly identified in 2010 and 2022 using Deep Neural Network (DNN) based on multi-source geographical data. Then, seven typical ESs were assessed using multiple models, and their interactions were examined through correlation analysis, coupling coordination degree model, and a self-organizing feature mapping network approach. At last, this study highlighted the complex responses of ESs relationships to dramatically changing spatial structure of urban-fringe-rural areas and proposed landscape management strategies. The findings include the following: (1) from 2010 to 2022, the spatial structure of urban-fringe-rural areas in Suzhou changed considerably, with 69.04% rural areas transformed into fringe areas, and 50.83% fringe areas developed into urban areas; (2) based on transition process, the region was further divided into urban maintenance, urban expansion, fringe maintenance, fringe expansion, and rural retention areas. Most of the mean value of ESs showed a gradient increasing differences along urban-fringe-rural, while the greatest decrease occurs in fringe expansion and urban expansion areas; and (3) interactions for changes in ES pairs also more closely linked in these two regions, with synergies dominating. The coupled coordination index among multiple ESs declines significantly in these areas, degrading from key coordination to key or mild trade-offs bundles. The results show ES interactions exhibit significant spatial variability under the evolution of metropolitan spatial structure, thus innovatively proposing integration of ESs synergies into urban-fringe-rural development framework to support overall landscape sustainability.

## Introduction

The traditional urban-rural dichotomy has long defined the territorial structure of cities and rural areas. However, with urbanization, increasing interactions between urban and rural elements have blurred these boundaries, especially in metropolitan areas [1–3]. Scholars proposed the existence of a vast, transitional, dynamically evolving zone between urban-rural areas as the urban-rural fringe [4,5]. Scott emphasized that the fringe area is a distinct geographic unit, characterized by its evolving nature [6]. Thus, the urban-fringe-rural geographic classification model is gradually replacing the original dichotomy to capture complexities of regional spatial organization. However, most studies have focused on urban expansion, exploring its identification, evolutionary process, and impacts [7–10]. Less attention has been paid to broader spatial structure of urban-fringe-rural areas and their associated impacts. Properly defining the spatial extent of these areas is crucial for understanding territorial structures, assessing the socio-economic and spatial consequences of their evolution, optimizing land use, and enhancing ecological protection.

Urban areas are characterized by high population densities and intensive industrial activities, with land use dominated by built environments [11,12]. Rural areas, in contrast, feature lower population densities and limited industrial development, with land predominantly devoted to agriculture and natural ecosystems [13]. The fringe areas, situated between urban and rural areas, exhibit a transitional mix of geographic elements [14,15]. This regional geospatial structure reflects the distribution of social and economic factors [16], with shifts symbolizing changes in human activities that impact ecosystem services (ESs). ESs are the benefits humans directly or indirectly derive from ecosystems, such as regulating, provisioning, and cultural services [17,18]. The interactions among ESs can involve trade-offs, where the increase of one service reduces another, or synergies, where multiple ESs are enhanced simultaneously [19,20]. This variability makes landscape less capable of providing stable, long-term ESs, which in turn affects human well-being [21,22]. Efforts to enhance ES synergies between urban and rural areas are gain attention, involving ES assessments, landscape fragmentation, ecological network security, and multifunctional ecological agriculture [23–26]. However, many studies separately focus on urban or rural areas, often overlooking the unique dynamics of the fringe zone.

Megacities, characterized by large populations and expansive urbanization, present a complex urban-fringe-rural spatial structure [27]. The concentration of population and industry in these cities leads to significant, often irreversible land-use changes. These shifts pose potential risks to ESs and their trade-offs, further intensifying urban ecological unsustainability [28–30]. the evolution of urban-fringe-rural spatial structures helps clarify the complexity of urban-rural systems and their dynamic patterns. By examining how these changes affect ESs, it is possible to more accurately assess and predict ES supply, changes, and interactions, leading to more effective strategies for optimizing and protecting these services and promoting sustainable urban-rural development. Hence this study makes efforts to address the following questions: What are the spatiotemporal patterns of urban-fringe-rural structural evolution in megacities? How do ESs trade-offs and synergies respond to these changes? How can ES synergies be integrated into sustainability planning for urban–fringe–rural landscapes?

**Fig 1:**
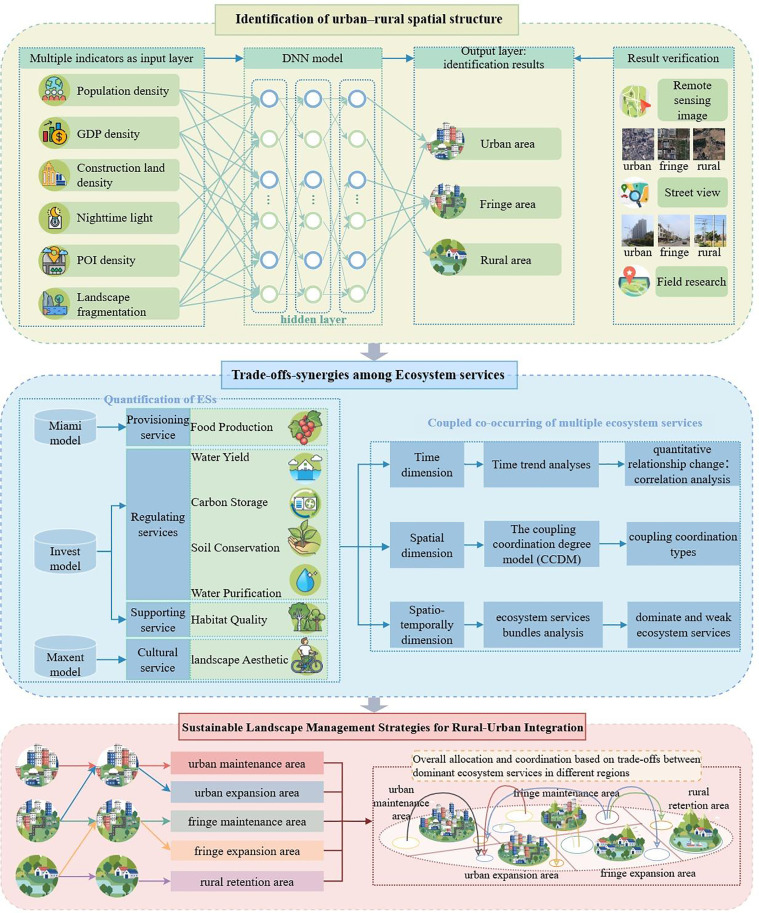
Flow chart of the study.

This study takes Suzhou—a typical megacity in China—as an example. Over the past decade, Suzhou has experienced rapid urban growth, especially at the urban fringe, resulting in significant ecological challenges. As such, it provides an ideal setting for investigating the evolution of urban-rural spatial structures and the performance of ESs trade-offs. In this study, a deep neural network (DNN) model was constructed to identify urban, fringe, and rural areas in Suzhou, subdividing the territory into five zones based on transformation trends from 2010 to 2022. Then 7 key ESs were assessed by integrating models like InVEST and Maxent. Subsequently, correlation analysis, coupled synergies, and clustering technique were applied to explore the spatiotemporal dynamics of ESs relationships. The results help reveal the evolutionary patterns of ESs across these geographic zones. Finally, refined landscape management strategies are proposed. By mapping and prioritizing areas of intense ES trade-offs, integrating ES synergies into the classification and planning of urban-fringe-rural landscapes, planners can employ targeted interventions, such as green infrastructure, multifunctional agriculture, or ecological corridors. This can provide a clear pathway to sustain overall landscape functionality, balance competing land-use demands, and strengthen regional resilience. The flowchart of this study is shown in Fig 1.

## Literature review

### Urban–fringe–rural identification and evolution

Within the longstanding traditional framework of the urban-rural dichotomy, previous studies often distinguish urban and rural areas based on multi-source data like land-use data, nightlight intensity, point of interest (POI), and common research topics include urban sprawl, land-use transition, ESs change, and environmental degradation [31–34]. With a deeper understanding of urban fringe areas, the urban-fringe-rural triadic structure has gained increasing attention from scholars, gradually replacing the urban-rural binary structure as the regional classification framework for theoretical and empirical research in fields such as geography, urban planning, land resource management, and ecological planning [35,36]. Mutation detection and comprehensive evaluation methods are two main models to identify urban, fringe, and rural areas. The former uses clear spatial data like POI and nighttime lights to find spatial differentiation [37], while the latter relies on socio-economic indicators and spatial clustering [38,39]. Through informative, these methods are time-consuming and resource-intensive, with limited scalability in spatiotemporal analysis. Recent advances data processing and artificial intelligence have led to the application of machine learning methods. For example, Liu employed deep neural networks (DNNs) to identify urban-fringe-rural regions based on POI data [40], but this approach’s reliance on single-dimensional data limits its ability to capture the complex characteristics of urban-fringe-rural transitions. On the other hand, Jiao utilized graph convolutional networks (GCNs) with multi-source data to analyze spatial changes over time [41], but it primarily focused on scale variations, lacking a deeper exploration of the dynamic evolution of spatial features.

To fill this gap, this study employs a DNN model to identify the spatial boundaries of urban, fringe, and rural areas. The DNN model is a type of artificial neural network that consists of multiple layers, supporting to learn complex representations of multi-source geographic data. It is characterized by its ability to handle large, high-dimensional datasets and model non-linear relationships, making it ideal for classifying spatial units into urban, fringe, and rural types. After identifying the spatial extent of these areas at two temporal nodes for a typical megacity, this study further explores the spatiotemporal evolution of the urban-fringe-rural regional structure. Additionally, it conducts a thorough analysis of the ESs relationship in the context of these spatial changes.

### ESs trade-off and synergies evaluation and influencing factor analysis

ESs synergy has become a key objective in ecological planning and sustainable development, as it optimizes ecological functions and supports ecosystem health [42]. ESs are typically classified into provisioning, regulating, supporting, and cultural services [43]. Their assessment often involves process-based models (e.g., InVEST, SWAT) or statistical models (e.g., regression, time series analysis), using ecological principles, historical data, and GIS tools [44,45]. To understand the complex interactions between ESs, correlation analysis and spatial models are commonly employed, revealing trade-offs or synergies [46]. Factors influencing ES trade-offs include land-use, urbanization, climate change, ecosystem configuration, and socio-economic drivers such as population density and agricultural intensity [47–49]. Strategies for optimizing ES synergies generally focus on integrated land management, multifunctional land uses, ecosystem connectivity, and sustainable agricultural practices [50,51]. While research on ES trade-offs has primarily examined urban expansion, studies show that urban growth tends to reduce provisioning services (e.g., agricultural output, resource depletion) but can enhance cultural (e.g., recreation) and regulating services (e.g., urban climate regulation) [52]. Some studies also explore ES dynamics in urban fringe areas, highlighting competition among ESs due to land-use changes [53]. Others have balanced ES from a comprehensive rural-urban distribution perspective, indicating that rural areas prioritize ecological services like water retention and biodiversity, while urban areas emphasize cultural and regulating services [26,54]. However, there has been limited analysis of how ES trade-offs and synergies evolve across the broader spatial structure of urban, fringe, and rural areas. This gap limits the ability to manage ESs in a dynamic, holistic, and territorially comprehensive framework.

The evolution of urban-fringe-rural spatial structures, especially in metropolitan regions, is widespread and continuous [55]. Once rural areas transition to urban or fringe zones, reversing this transformation is uncommon, as developed land seldom reverts to its non-built state. This irreversibility underscores the long-term consequences of land use changes. The urban-fringe-rural structure can be divided into five zones, including urban maintenance zones, urban expansion zones, fringe maintenance zones, fringe expansion zones, and rural retention zones. Each zone exhibits unique ES characteristics, encompassing both trade-offs and synergies. Understanding how ES relationships respond to dynamic changes in these zones is crucial for advancing sustainable development and optimizing ES management across urban and rural landscapes, helping to balance ecological integrity with the development needs of megacities.

## Study area and data source

**Fig 2:**
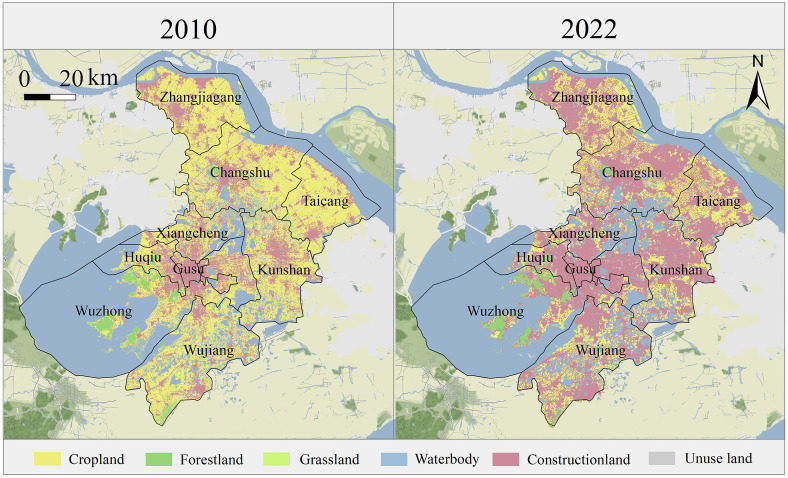
Land use map in Suzhou (2010 and 2022) based on GlobeLand30 classification. (Republished from Ministry of Natural Resources of China, http://bzdt.ch.mnr.gov.cn).

Suzhou (30° 47’–32° 02’ N, 119° 55’–121° 20’ E) is a prefectural city in Jiangsu Province, Southeast China, with a GDP of 2,395.83 billion yuan (333.28 USD) by the end of 2022, ranking among the top 20 regions in China. The city has a population of 12.911 million and an urbanization rate of 74.42%, making it a typical megacity selected for this case study. The rapid expansion of construction land (Fig 2) has significantly altered the urban-rural landscape. This growth, particularly evident in fringe areas where farmland and wetlands are being converted, creates competing demands on ESs—for instance, boosting housing and transportation capacity while reducing flood regulation and biodiversity. These intertwined trade-offs highlight the need to precisely map how different zones (urban core, expanding fringe, and rural hinterlands) contribute to ESs relationships. By understanding which areas see the sharpest conflicts between development and ecological functions, planners can target conservation efforts where they matter most, such as protecting critical wetlands in fast-urbanizing corridors or promoting green infrastructure in newly developed suburbs.

In this study, spatial data and statistical records were used to quantify ESs. All data were reprojected to a consistent coordinate system, and the spatial resolution was harmonized to 30 m. ES trade-offs were assessed at the village administrative unit level based on grid-scale measurements. Following were main types of data and descriptions (Table 1).

**Table 1:** Data sources and descriptions.

Data Type	Formats/Resolution	Description & Source
**Administrative boundary**	Shapefile	Resource and Environment Science and Data Center (https://www.resdc.cn)
**Digital Elevation Model**	Raster, 30 m	Geospatial Data Cloud (http://www.gscloud.cn)
**Land cover**	Raster, 30 m	GlobeLand30 (http://www.globallandcover.com/)
**Annual average precipitation**	Raster, 1 km	National Meteorological Information Center (http://data.cma.cn)
**River basin**	Shapefile	Extracted from DEM data (www.resdc.cn)
**Evapotranspiration**	Raster, 30 arc-seconds	FA0–56 Penman–Monteith ET0 data (https://cgiarcsi.community)
**Soil properties**	Raster, 1 km	Soil data from China Soil Map and Census (www.resdc.cn)
**NDVI**	Raster, 30 m	SPOT/VGT (20m) and MODIS NDVI (500m) fused using STARFM spatiotemporal fusion algorithm. Resampled to 30m via cubic convolution (www.resdc.cn)
**Population density**	Raster, 1 km	China Population Distribution Dataset of the Resource and Environmental Sciences Data Registry and Publishing System (http://www.resdc.cn/DOI)
**Nighttime-light**	Raster, 500 m	CALibrated NTL Dataset (CALNT v2.1) [56], a global NPP-VIIRS night-like light dataset for 2000–2022 generated by a self-coder-based cross-sensor calibration scheme in the coordinate system GCS_WGS_1984
**GDP**	Raster, 1 km	China GDP Spatial Distribution Dataset (http://www.resdc.cn/)
**Point of Interest (POI)**	Shapefile	Data from Baidu Open Platform (http://lbsyun.baidu.com/) using Python 3.11

## Methodologies

### Identification of urban–fringe–rural spatial structure

The first challenge in classifying urban, fringe, and rural areas lies in establishing objective criteria for training the DNN model and validating the results. Given the absence of standardized frameworks, prior studies have relied on manual identification. In this study, five experts in urban planning and geography were engaged to identification, and following steps were taken: 1) Experts were divided into two groups—four in the identification group and one in the validation group; 2) For 2010 and 2022, 1000 random geographic points were generated for each year. Each point was input into Google Maps with a 1000-meter radius, and satellite images within this radius were extracted; 3) Two members of the identification group reviewed and labeled the images as rural (0), fringe (1), or urban (2), resulting in 2,000 labeled images; 4) If both members agreed on the label, it was forwarded to the validation group for final assessment. If the validation expert agreed, the label was confirmed; 5) In cases of discrepancies, the remaining identification group members re-evaluated the images, and the validation process repeated. If disagreement persisted, the point was discarded and a new one generated (Table 2).

**Table 2:** Example of manual identification.

Location	Identification group (1)	Identification group (2)	Validation group
**31.34°E,120.48°N**	2, 2	/	2
**31.36°E,120.45°N**	0,1	1,1	1
**31.55°E,120.88°N**	0,0	/	0

Building upon this foundation, two DNN classification models were constructed for 2010 and 2022, each trained with 1000 labeled sample points (80% training, 20% test). The model architecture included an input layer, two hidden layers (128 and 64 neurons), and an output layer with Softmax activation for a three-class classification. The model used the Adam optimizer with a learning rate of 0.001, and sparse categorical cross-entropy as the loss function. Accuracy, calculated as the ratio of correctly predicted samples to total samples, was the primary evaluation metric.

Key geographic features were selected to represent the distinctive characteristics of urban, fringe, and rural areas. These included population density, GDP density, construction land density, POI density, nighttime light brightness, and landscape fragmentation (Table 3). All datasets were processed at a 1 km spatial grid scale. Dropout layers (rate = 0.5) were added after each hidden layer to prevent overfitting. The model captured and learned complex nonlinear relationships between the input features using deep neural networks.

**Table 3:** DNN model input indicators for urban-fringe-rural classification.

Data Type	Formula	Description
**Population density**	–	Gradient from high agglomeration in urban to low in rural areas
**GDP density**	–	Total GDP per unit area; high in urban, low in rural areas
**Construction land density**	$\text{PLAND}=\frac{a_{i}}{A}\times \text{1}\text{0}\text{0}$ $a_{i}:$the area of construction land; *A* represents the total land use area	Reflects human activity intensity; urban areas have higher construction land density
**Nighttime-light**	–	Indicates economic vitality; higher in urban areas
**landscape fragmentation**	$W=-\sum_{n=\text{1}}^{N}X_{n}\text{ln}(X_{n})$ *W* denotes landscape fragmentation, $X_{n}$ represents the proportion of a particular land type in a grid, and *n* is the number of land use types in the same grid.	Measures habitat fragmentation; urban fringe areas, where natural and semi-natural habitats are disrupted by human activities, show high fragmentation
**Point of Interest (POI)**	$F_{i}=\frac{n_{i}\times w_{i}}{\sum_{i=\text{1}}^{m}\left(n_{i}\times w_{i}\right)}\times \text{1}\text{0}\text{0}\%$ $F_{i}$is the urban function assessment based on POI types, where $n_{i}$ is the kernel density of the ith POI type, and $w_{i}$ is its weight, derived from hierarchical analysis.	Reflects urban functional services; POI density decreases from urban to rural areas. This study uses kernel and weighted summation methods to assess urban functions per unit area, considering the distribution densities of various types

### Quantifying ESs

Guided by the principles of differences in ESs in urban and rural landscapes, the importance of ESs to urban and rural residents, and data availability, seven ESs, including one provisioning service (food production), four regulating services (carbon storage, soil conservation, water purification, and water yield), one supporting service (habitat quality), and one cultural service (landscape aesthetics), were selected in this study during 2010 and 2022. All ESs were quantified at a grid spatial resolution of 30 meters. The detailed assessment methodology is presented in Table 4.

**Table 4:** Methodologies and principles for assessing ecosystem services (ESs).

ESs	Methods	Formula	Description
**Food Production (FP)**	Thornthwaite Memorial and Miami models [57]	$W_{T}=\text{3}\text{0}\text{0}\text{0}\text{0}/(\text{1}+e^{\text{1}.\text{3}\text{1}\text{5}-\text{0}.\text{1}\text{1}\text{9}T})$ $W_{R}=\text{3}\text{0}\text{0}\text{0}\text{0}/(\text{1}-e^{-\text{0}.\text{0}\text{0}\text{0}\text{6}\text{6}\text{4}R})$ $W_{v}=\text{3}\text{0}\text{0}\text{0}\text{0}/(\text{1}-e^{-\text{0}.\text{0}\text{0}\text{0}\text{9}\text{6}\text{9}\text{5}(v-\text{2}\text{0})})$ $V=\text{1}.\text{0}\text{5}R/[\text{1}+{{\left(\text{1}.\text{0}\text{5}R/L\right)}^{\text{2}})]}^{\text{1}/\text{2}}$ $L=\text{3}\text{0}\text{0}+\text{2}\text{5}T+\text{0}.\text{0}\text{5}T^{\text{3}}$ *W* $=min(W_{T},W_{R},W_{V})$ $Y_{i}=W_{i}\times K_{i}$	*T*:mean annual temperature(°C); *R*: annual precipitation (mm); *L*:mean annual maximum evapotranspiration(mm); *V*:mean annual actual evapotranspiration(mm);$W_{T}$、$W_{R}$、$W_{V}$ are the vegetation production potential determined by the mean annual temperature, annual precipitation, and evapotranspiration respectively (kg/ha·a); *W*: standard climatic production potential of study area.$Y_{i}$: integrated production potential of plot *i*; $W_{i}$:standard climatic production potential; $K_{i}$: integrated revision coefficient of production potential of plot *i*.
**Soil Conservation (SC)**	InVEST “SDR” model [58]	SC=R×K×LS×(1−C×P)	SC:soil conservation, $\text{t}/{\text{km}}^{\text{2}}$, R:rainfall erosivity, $\text{MJ}{\text{mm}{\text{hm}}^{-\text{2}}{\text{h}}^{-\text{1}}\text{a}}^{-\text{1}}$, K:soil erodibility, $\text{t}{{\text{h MJ}}^{-\text{1}}\text{mm}}^{-\text{1}}$, C:vegetation cover factor, dimensionless; P:soil and water conservation factor, dimensionless; LS: factor of slope length and gradient, dimensionless.
**Water Yield (WY)**	InVEST “Water Yield” model [59]	$Y_{\left(xj\right)}=\left(\text{1}-\frac{{AET}_{\left(xj\right)}}{P_{\left(x\right)}}\right)\times P_{\left(x\right)}$	$Y_{\left(xj\right)}/{AET}_{\left(xj\right)}:$annual water yield/ annual actual evapotranspiration (mm) on grid *x* for land use type *j*;$P_{\left(x\right)}$:annual rainfall (mm) on grid *x*; ${PET}_{\left(x\right)}$:potential evapotranspiration.
**Carbon Storage** **(CS)**	InVEST “Carbon Storage” model [60]	$C_{tot}=C_{above}\cdot C_{below}{\cdot C}_{soil}\cdot C_{dead}$	$C_{tot}$: total carbon stock (t), $C_{above}$、$C_{below}$:carbon in above- and below-ground biomass, $C_{soil}:$carbon in soil, $C_{dead}$:carbon in dead organic matter.
**Nitrogen Output (TN)/ Phosphorus Output (TP)**	InVEST “NDR” model [61]	${ALV}_{x}={HSS}_{x}\times {pol}_{x}$	${ALV}_{x}$: nutrient output value of corrected grid *x*; ${pol}_{x}$:nutrient export coefficient of grid *x*; ${HSS}_{x}$:hydrological sensitivity score of grid *x*; The capacity of water quality purification is inversely proportional to magnitude of nitrogen and phosphorus output coefficients.
**Habitat Quality (HQ)**	InVEST “Habitat Quality” model [62]	$Q_{\left(xj\right)}=H_{j}\times \left(\text{1}-\frac{{D_{\left(xj\right)}}^{\text{2}}}{{D_{\left(xj\right)}}^{\text{2}}+k^{\text{2}}}\right)$	$Q_{\left(xj\right)}:$habitat quality of grid *x* in land use type *j*; $H_{j}$:habitat adaptability of grid *x* in land use type *j*; $D_{\left(xj\right)}$:degree of habitat degradation of grid *x* in land use type *j*; *k*:half-saturation constant. The values range from 0 to 1.
**Landscape Aesthetics (LA)**	MaxEnt model [63,64]		Using POI data combined with environmental variables, including natural and man-made condition indicators: slope, distance from water bodies, elevation, land use and distance from roads.

### Analyzing the trade-off and synergy relationships among ESs

Correlation analysis, coupling coordination degree model, and self-organizing mapping network methods were employed to investigate interactions between multiple ESs across temporal, spatial, and spatiotemporal dimensions.

#### Correlation analysis.

To eliminate scale differences between ESs, Min–Max normalization was applied to standardize the data:


$${ES}_{std}=\frac{{ES}_{obs}-{ES}_{min}}{{ES}_{max}-{ES}_{min}},$$ (1)

where ${ES}_{std}$ is the normalized ES value, ${ES}_{obs}$ is the observed value, and ${ES}_{min}$ and ${ES}_{max}$ are the minimum and maximum observed values.

Spearman’s nonparametric correlation analysis was then used to identify trade-off or synergy relationships among ES changes from 2010 to 2022. Positive correlations indicate synergy, while negative correlations indicate trade-offs. The “corrplot” package in R4.3.1 was used to visualize correlations among ESs in 2010 and 2022. The correlation coefficient was calculated as:


$$R_{\left(xy\right)}=\frac{\sum_{n=\text{1}}^{n}\left(X_{ij}-\overset{―}{X})(Y_{ij}-\overset{―}{Y}\right)}{\sqrt{\sum_{n=\text{1}}^{n}{\left(X_{ij}-\overset{―}{X}\right)}^{\text{2}}}\sqrt{\sum_{n=\text{1}}^{n}{\left(Y_{ij}-\overset{―}{Y}\right)}^{\text{2}}}},$$ (2)

where $R_{\left(xy\right)}$ is the correlation coefficient (value range [−1,1]), and $X_{ij}$ and $Y_{ij}$ are ES data values.

#### Coupling coordination degree model (CCDM).

To explore the interactions among multiple ESs, we employed the coupled coordination degree model (CCDM) at the village scale to assess coordination changes in Suzhou from 2010 to 2022. Spatial trend analysis identified shifts in incoordination, shedding light on the evolutions of ES relationships over time and space.

Coupling measures the interaction strength between systems, quantified by the coupling degree (CD) [65]. The coupling coordinated degree (CCD) reflects the level of integrated system development. For *k* systems, the coupling degree model (CDM) is given as:


$$C={\left\{\left(U_{\text{1}}\times U_{\text{2}}\times \cdots \times U_{k}\right)/\left[\prod_{\text{1}\leq i,j\leq k,i\neq j}U_{i}+U_{j}\right]\right\}}^{\text{1}/k},$$ (3)

where $U_{i}$represents the average value of each ES in a village, and *C* ranges from 0 (low interaction) to 1 (high interaction). When *C* = 1, the systems are fully integrated, and When *C* = 0, they are independent [66]. To assess coordination, the CCDM formula is:


$$T=aU_{\text{1}}+bU_{\text{2}}+cU_{\text{3}}+dU_{\text{4}}+eU_{\text{5}}+fU_{\text{6}}+gU_{\text{7}}+hU_{\text{8}}(i\neq j),and$$ (4)

$$D=\sqrt{C\times T},$$ (5)

where *T* reflects the overall impact of the ESs, and *a*, *b*, *c*, …, *h* are coefficients that reflect the relative importance of each service (values: 0.12 for most services, and 0.16 for habitat quality, based on expert recommendations). *D* ranges from 0 to 1, with higher values indicating better coordination. Coordination is categorized into six levels (Table 5), from extreme incoordination (F) to high-quality coordination (A) [67].

**Table 5:** Coupling coordination types.

Category	D Value	Description	Interval
**Synergy**	(0.8,1.0]	High-quality coordination	A
	(0.6,0.8]	Good coordination	B
	(0.5,0.6]	Low coordination	C
**Trade-off**	(0.4,0.5]	Mild incoordination	D
	(0.2,0.4]	Moderate incoordination	E
	[0,0.2]	Extreme incoordination	F

#### Self-organizing feature mapping network.

To capture the response of key ESs and their trade-offs within the urban-fringe-rural territorial structure evolution, Self-Organizing Map (SOM) clustering was applied. SOM is an unsupervised neural network that reduces dimensionality, visualizing high-dimensional data in a 2D grid while preserving topological relationships [68]. This method identifies patterns in ES interactions and their trade-offs, combining principal component analysis (PCA) and K-means clustering. SOM maintains the topology of input space through nearest-neighbor relationships and incorporates spatial data. Its ability to handle large, complex datasets while remaining interpretable makes it well-suited for exploring spatially distributed ESs across diverse structures. Additionally, SOM’s competitive learning algorithm detects hidden relationships without prior labeling, making it flexible for analyzing non-linear interactions among multiple ESs in dynamic environments [69]. In this study, the urban fringe of Suzhou was used as input, and the optimal number of clusters was determined by calculating the Davies–Bouldin index for 2–15 classes using the “kohenen” package in R4.3.1.

## Results

### Identification of urban–fringe–rural spatial structure and its evolution

**Fig 3:**
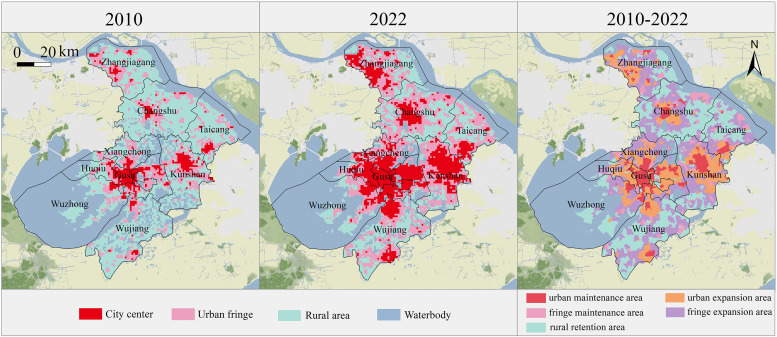
Spatiotemporal patterns of urban–fringe–rural structure in Suzhou. (Republished from Ministry of Natural Resources of China, http://bzdt.ch.mnr.gov.cn).

This study maps the urban–fringe–rural spatial structure of Suzhou in 2010 and 2022, achieving DNN model accuracies of 0.9502 and 0.8850. The results (Fig 3) show that urban fringe areas are primarily located in the central and northern regions, with the south remaining predominantly rural. Both the central city and fringe areas exhibit polycentric dispersion and outward expansion. By 2022, the urban fringe covered 40.42% of the area, marking a 75.83% increase, while urban areas grew by only 2.13%. Conversely, rural areas decreased by 46.23%, covering 1,686 km^2^. Transfer analysis indicates that most land conversions occurred from rural to fringe areas (1,713 km^2^) and from fringe to urban areas (862 km^2^), representing 69.04% and 50.83% of the respective areas by 2022. This highlights that while the urban fringe is rapidly expanding, the rate of its transition to urban areas is slower, potentially leading to land resource inefficiency.

At the county level, there is notable spatiotemporal heterogeneity. Gusu District, Industrial Park, Huqiu District, and Xiangcheng District have the highest fringe-to-urban ratios (over 90%), indicating strong urbanization pressures. Changshu, Wujiang, and Taicang showed the fastest fringe growth (over 20% from 2010 to 2022), while Huqiu and Industrial Park experienced the most rapid urban growth (over 38%). Central Suzhou is characterized by urban expansion, while the northern and southern regions show more fringe expansion, reflecting differentiated urbanization patterns. Wujiang and Taicang’s substantial rural areas suggest lower urbanization, indicating potential for future development.

Transformation scenarios were classified into five types, namely urban maintenance area (sustained urban from 2010 to 2022), urban expansion areas (transformed from fringe to urban), fringe maintenance area (sustained urban fringe), fringe expansion area (transformed from rural to urban fringe), and rural retention area (sustained as rural). Areas transitioning from urban or fringe to rural were excluded due to their minimal proportion, indicating that urbanization of rural areas is typically irreversible. fringe expansion areas (34.36%) make up the largest share, followed by rural retention (31.42%) and urban expansion (22.09%) (Table 6).

**Table 6:** Transfer matrix of urban–fringe–rural spatial structure in Suzhou, 2010–2022.

2010-2022	Rural area	Fringe area	City area
**Rural area**	44.89%	47.46%	7.65%
**Fringe area**	0.78%	38.04%	61.18%
**City area**	0.00%	4.82%	95.18%

### Spatiotemporal heterogeneity of ESs across urban–fringe–rural areas

**Fig 4:**
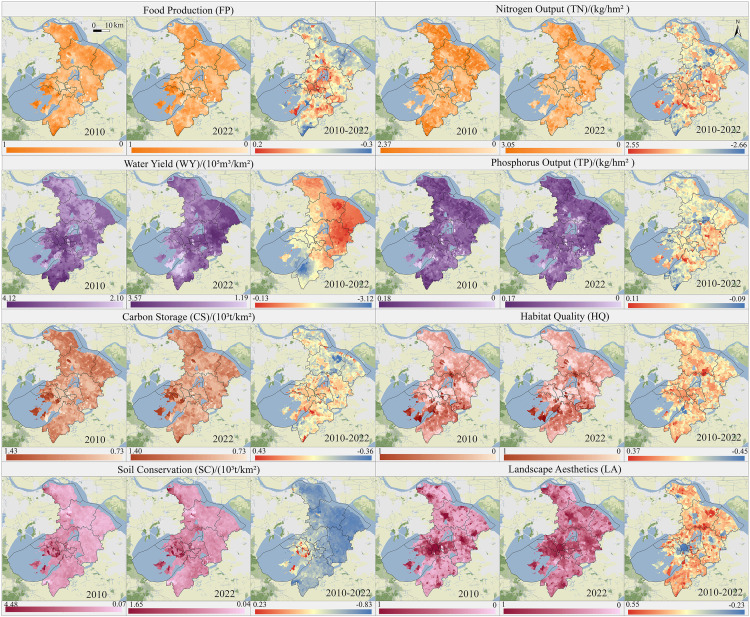
Spatial patterns and changes of ESs in Suzhou from 2010 to 2022. (Republished from Ministry of Natural Resources of China, http://bzdt.ch.mnr.gov.cn).

From 2010 to 2022, most ESs showed higher values in the northern and southern regions of Suzhou, with lower values in the central area. This trend applied to services such as carbon storage, water purification, soil conservation, and habitat quality (Fig 4). Food production was highest in the northeastern and southwestern regions, linked to arable land, and lowest in mountainous and urban areas. Water yield and landscape aesthetics both peaked in the urban center. Overall, most services, except cultural services, declined, with food production declining by 37.68%, primarily in the urban core and northern fringe. Regulatory and supporting services significantly decreased near urban centers, while mountainous regions saw increases in water purification and soil conservation. Notably, water yield rose significantly, while habitat quality declined in the urban fringe.

**Fig 5:**
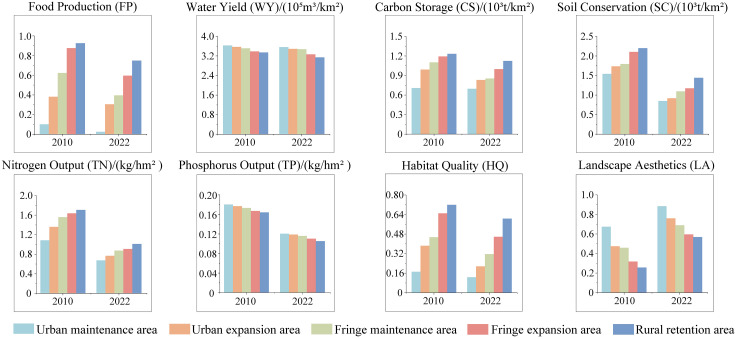
ESs Changes in urban, fringe, and rural areas from 2010 to 2022. (Republished from Ministry of Natural Resources of China, **http://bzdt.ch.mnr.gov.cn**).

To understand the response of ESs to urban-fringe-rural spatial structures, changes across five regions were counted and compared (Fig 5). Most ESs—such as food production, carbon storage, soil conservation, nitrogen output, and habitat quality—were highest in rural retention areas, followed by fringe expansion areas, and lowest in urban maintenance areas, showing a decreasing gradient with urbanization. Conversely, urban maintenance areas exhibit the highest mean values for water yield and landscape aesthetics, with elevated phosphorus output in urban expansion areas. From 2010 to 2022, urban expansion, fringe expansion and urban fringe areas experienced substantial decreases in food production, water yield, and carbon storage, alongside the largest increases in landscape aesthetics. Soil conversation fluctuated in these regions. Notably, food production, carbon storage, nitrogen-phosphorus output, and habitat quality declined most in fringe expansion areas, with nitrogen output, food production and habitat quality dropped by 46.33%, 36.57% and 30.52%, respectively. Meanwhile, landscape aesthetics increased most in rural retention areas.

### Trade-offs and synergies among ESs across urban-fringe-rural geographical structures

#### Correlation coefficients of ES pairs.

**Fig 6:**
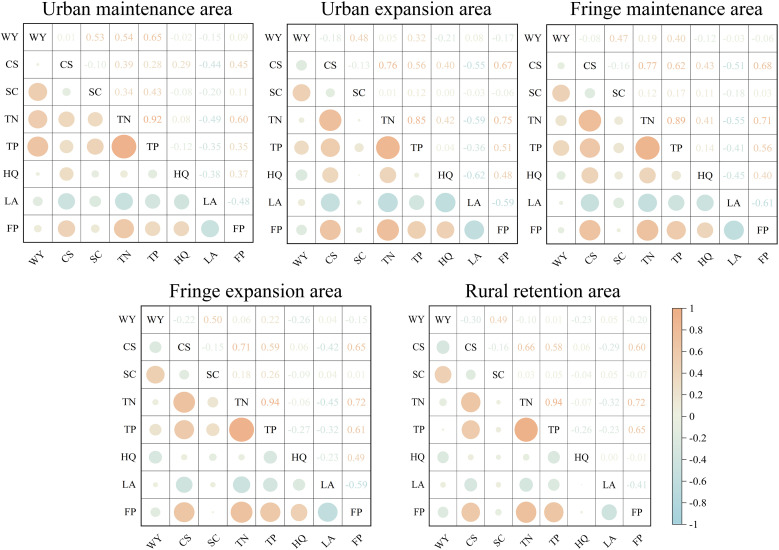
Correlation coefficients of ESs pairs. * FP—Food production; CS—Carbon storage; WY—Water yield; HQ—Habitat quality; SC—Soil conservation; TN—Nitrogen output; TP—Phosphorus output; LA—Landscape aesthetics.

The correlations between changes in ES pairs from 2010 to 2022 were calculated across five regions (Fig 6). Positive correlations indicate synergies, where two ESs increase or decrease together, while negative correlations reflect trade-offs, where one ES increases and the other decreases. The absolute value of the correlation coefficient indicates the strength of the relationship.

Overall, the correlation coefficients of most ES pairs grew stronger from 2010 to 2022, particularly as fringe or rural areas transitioned to urban or fringe areas. Trade-offs were most evident in urban and fringe expansion areas, particularly between food production and landscape aesthetics, habitat quality and landscape aesthetics, and carbon storage and water purification. Urban fringe areas, with their edge effects, showed stronger coupling of ESs under anthropogenic disturbance. In contrast, rural reserves mainly experienced a loss in both food supply and habitat quality, reflecting the ecological degradation associated with urbanization. Additionally, trade-offs between carbon storage and nitrogen/phosphorus export, habitat quality, and food production were observed across all regions.

#### Multiple ESs interaction relationships under CCDM.

**Fig 7:**
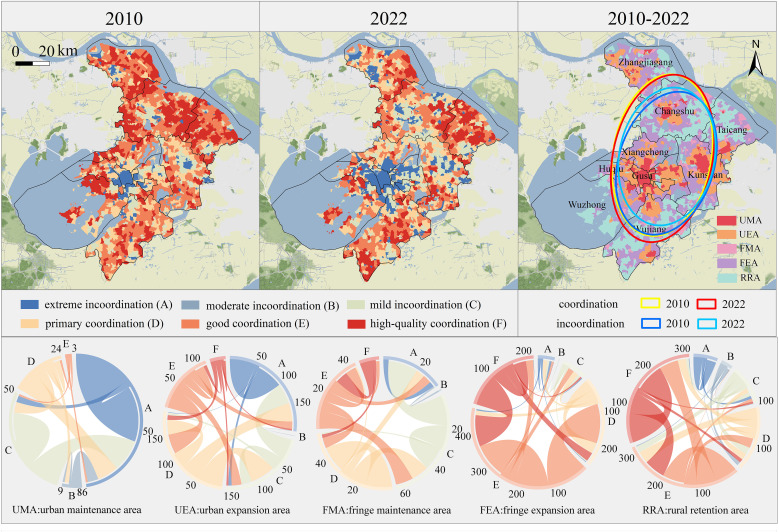
Multiple ESs interaction relationships under CCDM and trend direction analysis. (Republished from Ministry of Natural Resources of China, **http://bzdt.ch.mnr.gov.cn**).

To explore the interactions among multiple ESs in response to changes in urban-fringe-rural structures, a coupled coordination degree model was employed to Suzhou from 2010 to 2022. The results were categorized into six levels (Fig 7) and associated with the newly defined urban-fringe-rural territorial structure.

From 2010 to 2022, the average coupling coordination values for ESs in Suzhou decreased from 0.5820 to 0.4481, showing a shift from primary synergy to mild incoordination. ES interactions exhibit dissonance in urban maintenance areas but evolved toward harmony along the urban–fringe–rural gradient, reaching synergy in rural retention areas. The transfer matrix revealed that urban maintenance areas experienced growing dissonance, while rural retention areas shifted from mild dissonance to synergy, particularly in villages of southern Wujiang District, where ES relationships balanced. Notably, the most significant changes occurred in urban and fringe expansion areas. In urban expansion areas, ES relationships deteriorated from synergy to high dissonance, while fringe expansion areas saw a shift from good synergy to primary synergy.

These findings highlight the disparities in ES interactions during urban-fringe-rural transformation. Urban expansion areas faced greater risks of environmental incoordination, while fringe maintenance areas exhibited minimal change. Spatial analysis, supported by the standard deviation plot (Fig 7), shows that an expansion of incoordination areas and a reduction in synergistic ones by 2022. The directional trend of the plot spreads toward the northeast in line with fringe sprawl.

#### Interactions among multiple ESs.

**Fig 8:**
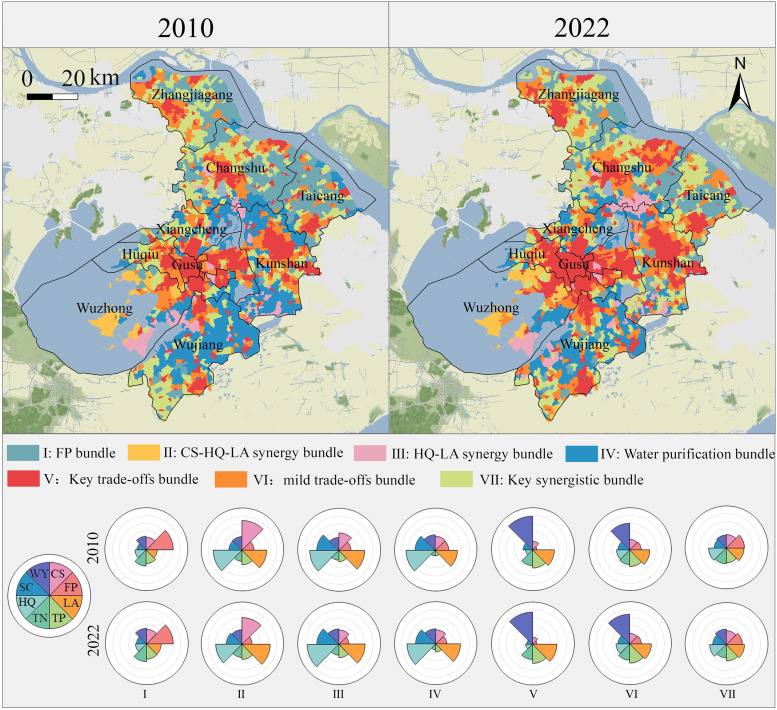
Spatiotemporal patterns of interactions among multiple ESs. (Republished from Ministry of Natural Resources of China, http://bzdt.ch.mnr.gov.cn). Composition and relative magnitude of ESs in the village scale. Longer segments represent higher ES supply. * FP—Food production; CS—Carbon storage; WY—Water yield; HQ—Habitat quality; SC—Soil conservation; TN—Nitrogen output; TP—Phosphorus output; LA—Landscape aesthetics.

This study utilized the self-organized mapping network clustering method to categorize ES bundles at the village scale, the smallest ecological unit in territorial spatial planning. Seven distinct ES bundles (Fig 8) were identified based on dominant services and their interactions: (1) *Key trade-offs bundle*. Found near urban areas, characterized by high water yield and landscape aesthetics but minimal food production and depleted other ESs. (2) *Mild trade-offs bundle*. Located in fringe areas, with high water yield and landscape aesthetics, but low food provisioning and reduced other ESs. (3) *Key synergistic bundle*. Primarily in rural areas, showcasing balanced ES provision and strong synergies. (4) *CS-HQ-LA bundle*. Found mainly in southwest rural Suzhou, with high carbon storage, and notable synergies among carbon storage, habitat quality, and landscape aesthetics. (5) *HQ-LA bundle*. Located in rural areas, dominated by landscape aesthetics and habitat quality, with strong soil conservation services. (6) *Water purification bundle*. Concentrated in areas with extensive water bodies (e.g., Wujiang District), offering effective water quality purification and low nitrogen/phosphorus outputs. (7) *FP bundle*. Primarily in northern Suzhou, dominated by high food production.

From 2010 to 2022, the urban maintenance area showed a dominance of key trade-offs, comprising 72.0% and 82.5% of the bundles, respectively. In urban expansion area, key trade-offs and mild trade-offs increased from 39.3% and 33.8% in 2010 to 52.6% for key trade-offs by 2022. Similarly, fringe maintenance areas exhibited a shift toward key trade-offs, from 34.6% and 30.7% in 2010 to 45.1% in 2022. In the fringe expansion area, the key synergistic bundle and water purification bundles were prominent in 2010 (39.2% and 25.2%), but by 2022, the area shifted to dominance by the key synergistic bundle and mild trade-offs (40.5% and 20.8%), while the water purification bundle declined to 17.8%. The rural retention area, initially dominated by food production, water purification, and key synergistic bundles in 2010 (26.9%, 21.2%, and 20.4%), but by 2022, saw these proportions decrease by 2022.

ES bundle transfers primarily occurred in the urban expansion, fringe expansion, and rural retention areas, with urban maintenance and fringe maintenance areas remained stable. In urban expansion areas, mild trade-offs shifted to key trade-offs due to increased built-up land, leading to degradation in soil and water conservation and habitat quality. The fringe expansion area exhibited varied ES bundle changes across regions. In central and northern regions, some villages near the urban fringe experienced an increase in water yield and a reduction in food production, transitioning from key synergistic bundles to mild trade-offs (19.4%). In Wujiang and Kunshan, certain villages saw a decline in water purification capacity, triggering cascading habitat quality trade-off effect that shifted them from water purification to mild trade-offs bundles (16.1%), while others transitioned to key synergistic bundles. In rural retention areas, notable changes occurred in the Taicang-Changshu border, where fragmented arable land diminished food production. Some villages near the fringe expansion area shifted from HQ-LA to mild trade-offs, indicating habitat quality degradation risk. Given the irreversible nature of urbanization, ES trade-off risks exhibit distinct stages, particularly in dynamically developing urban and fringe expansion areas.

## Discussion

### Evolution process of spatial structure for urban–fringe–rural areas in the metropolitan region

The delineation of urban-fringe-rural spatial structures through our DNN framework inherently balances methodological rigor and contextual adaptability. We employed city-wide random sampling (2,000 grids) and multi-stage expert validation (7.2% resampling rate) to minimize labeling bias while capturing Suzhou’s unique land use context. Specifically, we excluded large water bodies (>1 km^2^), which constitute 37.1% of the territory, and integrated fragmented forests (median size <0.5 km^2^) into the rural classification based on their inherently low-density characteristics (S1 Fig). When applying this framework to other contexts, researchers should be tailored to local land use profiles. For instance, implementing pre-classification masking for forest patches exceeding context-specific size thresholds (e.g., > 5 km^2^ in mountainous cities like Lishui or eco-cities like Xiamen) to avoid misclassifying densely forested areas as rural zones. Additionally, temporal validation across Jiangsu Province (2005–2022, accuracies: 0.84–0.95) through DNN and comparative analysis against traditional mutation detection methods (low accuracy: 0.66, S2 Fig) confirm the model’s capacity to decode gradient urbanization patterns characteristic of polycentric metropolises. These technical considerations underpin our core finding that Suzhou exhibits a multi-layered urban-fringe-rural structure, highlighting the continuous expansion of urban fringe areas in Suzhou. This pattern aligns with similar trends observed in other Chinese cities, such as Chengdu, Shanghai and Guangzhou [70–72]. Given the irreversible nature of rural-urban transitions and the inherent heterogeneity in these shifts of metropolitan cities, accurately capturing their dynamic evolution is crucial for sustainable landscape development.

**Fig 9:**
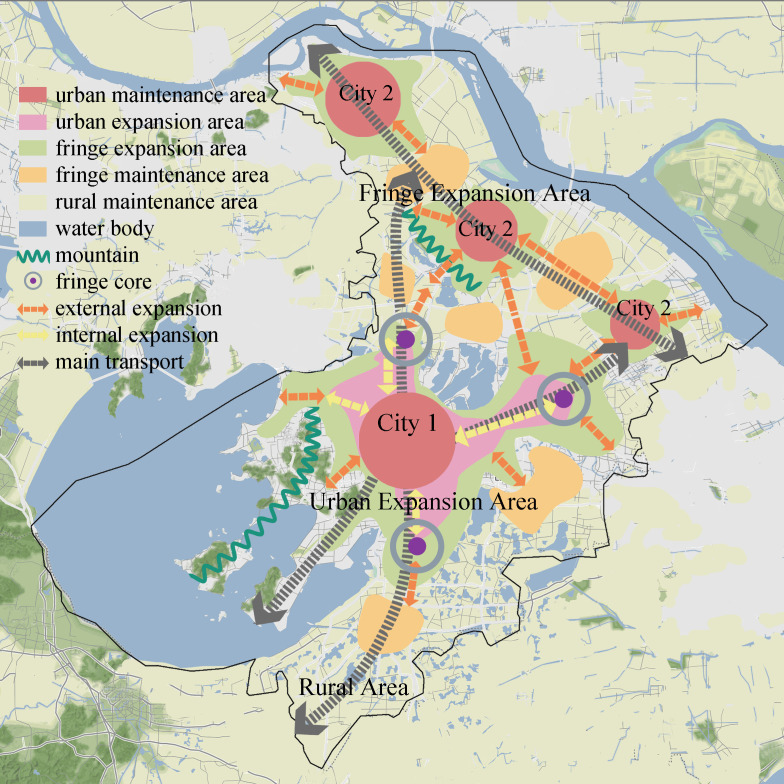
Structure diagram of dynamic urban–fringe–rural areas. (Republished from Ministry of Natural Resources of China, http://bzdt.ch.mnr.gov.cn).

The study categorizes the urban-fringe-rural continuum into five distinct types, revealing a multi-core developmental texture and differentiated formation mechanisms. In Suzhou, for example, the historical Gusu District (Fig 9, City 1) anchors the city’s urbanization trajectory. The “one core, four cities” policy has reinforced centralized growth, leading to simultaneous internal growth and external expansion across sub-regions like Xiangcheng, Huqiu, the Industrial Park, and Wujiang District. This phenomenon creates a unique multi-layered spatial structure, marked by overlapping urban, fringe, and fringe maintenance zones. Meanwhile, sub-city regions (City 2) predominantly exhibit external expansion, driven by transportation corridors and regional integration policies, influenced by Shanghai’s economic effect. These areas reflect decentralized urbanization, with fringe expansion zones along major transportation routes. This trend resonates with suburbanization patterns (e.g., suburban enclaves or exurban growth in metropolitan peripheries in the U.S. and Europe [73–75]), but also highlights the regional specificity shaped by China’s policy-driven urbanization.

The dynamic evolution of urban–fringe–rural spatial structures reflects a blend of internal densification and external expansion, offering a unique perspective for comparative studies on urban-fringe dynamics. This study also provides a practical foundation for exploring strategies for dynamic management, addressing social and ecological challenges, enhancing conservation, and promoting sustainable development in rapidly urbanizing regions.

### Changes in ESs trade-offs and their implications for urban–fringe–rural landscape sustainability

The dynamic evolution of urban–fringe–rural landscapes significantly influence ESs trade-offs and synergies, demonstrating the transitional and complex nature of these areas. Regulatory and supporting services like carbon storage, water purification, and habitat quality have markedly changed in fringe and expansion areas, intensifying trade-offs and driving the degradation of ES bundles. The most severe trade-offs are concentrated in rapidly urbanizing regions such as Kunshan and Changshu in northeastern Suzhou, where urban fringe expansion accelerates shifts from mild to severe trade-offs. This is driven by land-use changes and functional spillover effects from urban cores, like Gusu District. Some villages at the junction of Kunshan and the Industrial Park have undergone a leapfrogging development process, transitioning directly from key synergies to severe trade-offs between 2010 and 2022. This pattern underscores ecological risks and negative externalities associated with rapid urbanization in fringe areas.

Addressing these challenges requires integrating ES synergistic management into urban–fringe–rural sustainability planning. Urban fringe areas serve as critical ecological buffers, mitigating urban pressures. Protecting key ecological nodes—such as water sources and biodiversity hotspots—helps maintain landscape connectivity and reduce ecosystem degradation. For example, enhancing ecological corridors, as seen in the European Green Belt, facilitates species migration and gene flow, balancing ecological integrity with development. Additionally, the intensive land-use changes in urban fringe areas necessitate adopting green infrastructure strategies. The intensive land-use changes in urban fringe areas necessitate adopting green infrastructure strategies. Green belts, parks, wetlands, and ecological buffers can enhance ecological resilience and mitigate urbanization’s environmental impact. These measures can improve both environmental quality and residents’ quality of life. In Suzhou, targeted green development can address ecological trade-offs and support urban-rural integration. Finally, integrated planning is essential for the irreversible ecological and social transitions in fringe areas. Policies should emphasize urban-rural resource complementarities, leveraging rural areas for food supply and regulating services, while urban areas provide cultural and economic benefits. Equitable distribution of public services and infrastructure, including transportation and green spaces, can foster synergy between urban and rural systems, as demonstrated by international models. These strategies offer a balanced approach to sustainable socio-economic and environmental development.

### Limitations and future directions

While the proposed identifiable urban-fringe-rural framework effectively captures policy-driven metropolitan dynamics in Suzhou, its applicability to market-dominated cities or those with unique landscapes (e.g., mountainous or resource-depleted regions) requires further validation. Future efforts should prioritize two pathways: First, developing adaptive analytical modules tailored to different institutional contexts—for instance, incorporating land price indices in market-active areas and landslide risks in ecological corridors. Second, it is essential to engage stakeholders—including farmers, urban residents, government officials, and developers—in discussions about the findings and their views on ES trade-off decisions. Establishing a closed-loop system integrating “DNN identification-ES assessment-stakeholder engagement”, where resident preferences (collected through participatory platforms) directly inform model refinements, moving beyond post-hoc discussions. Finally, Additionally, the current semi-supervised model’s reliance on expert labeling could be optimized through disputed-area prioritization—focusing annotation efforts on ambiguous urban-rural transition zones rather than blanket sampling. This targeted approach maintains accuracy while enhancing scalability.

## Conclusion

In this research, multi-source geographic data and machine learning methods were emplyed to investigate the evolution of urban, fringe, and rural areas in Suzhou from 2010 to 2022. Key ESs and their interactions across urban–fringe–rural geospatial structures were analyzed, resulting in the identification of seven distinct ES bundles: key trade-offs bundle, mild trade-offs bundle, key synergistic bundle, CS-HA-LA bundle, HQ-LA bundle, water purification bundle, and FP bundle. These findings formed the basis for developing landscape optimization strategies to enhance synergies among ESs and promote sustainable urban-rural development.

This study contributes to the literature in two significant ways. First, the DNN model-based identification method provides a refined approach for delineating urban–fringe–rural spatial structures across various scales and timeframes in metropolitan regions. Second, by employing a case-study approach, an innovative framework is presented for recognizing, evaluating, and managing dynamically evolving ES interactions, offering valuable guidance for sustainable urban–fringe–rural landscape planning and governance.

## Supporting information

S1 Fig Spatial distribution of forests and water bodies in Suzhou. (JPG)

S2 Fig Spatial distribution of urban_fringe_rural areas in Suzhou. (JPG)

S1 File Data_ administrative boundaries/ annual precipitation grid/ average temperature per year/ dem/ land use/ Pawc/ population. (ZIP)

S2 File Data_GDP/ Ndvi. (ZIP)

S3 File Data_road. (ZIP)

S4 File Data_soil. (ZIP)

S5 File Excel_ESs and transfer matrix. (ZIP)

S6 File Results_urban-fringe-rural. (ZIP)
