# Supplementary figures and images for: Spatial structure evolution and ecosystem service relationship changes in urban-fringe-rural areas of megacities: Evidence from Suzhou, China
Source: PLoS One. 2025 Sep 24;20(9):e0332934. doi: 10.1371/journal.pone.0332934 (PMC12459847; doi:10.1371/journal.pone.0332934)

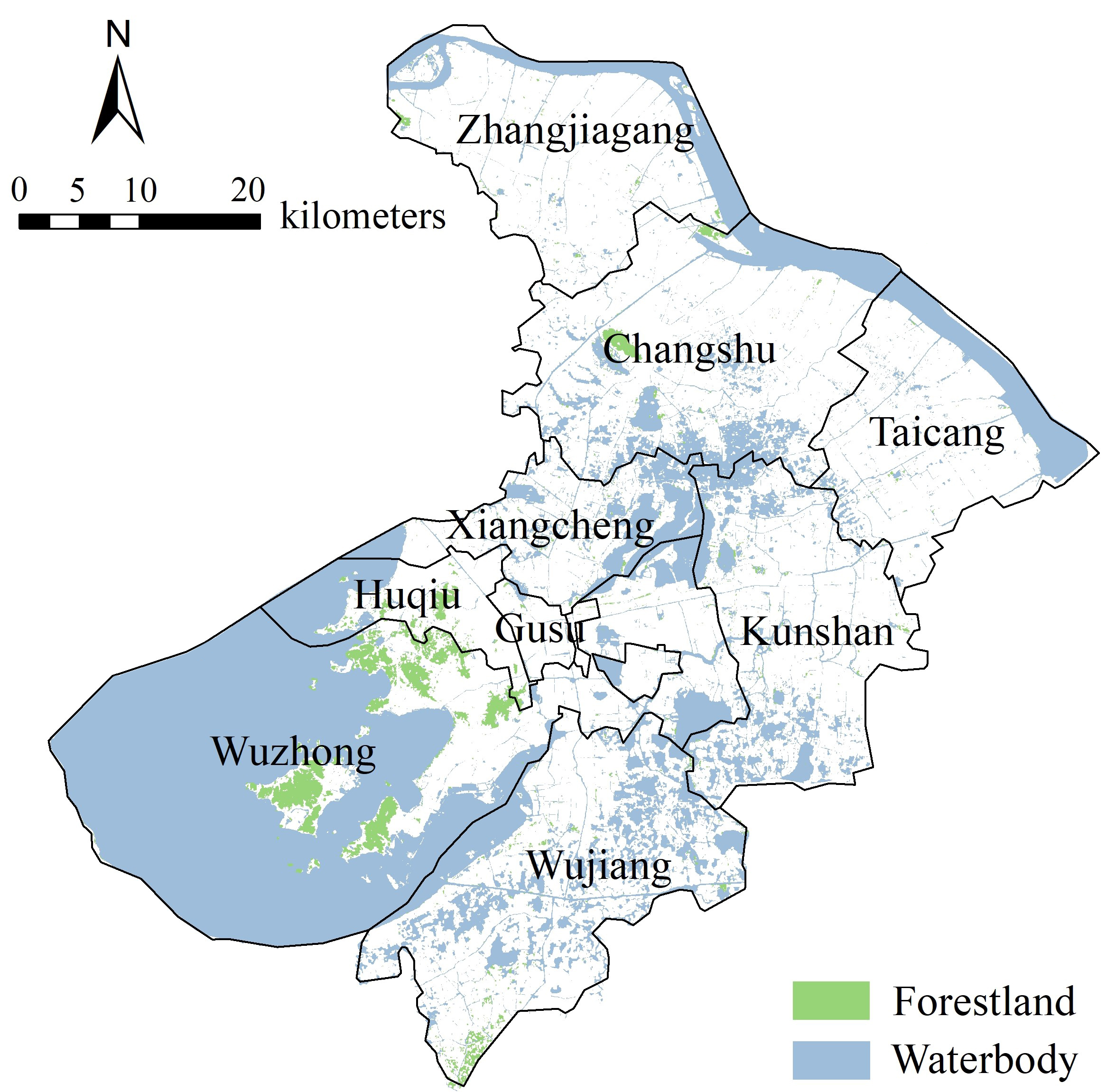

Supplement: S1 Fig — (JPG) [file pone.0332934.s001.jpg]

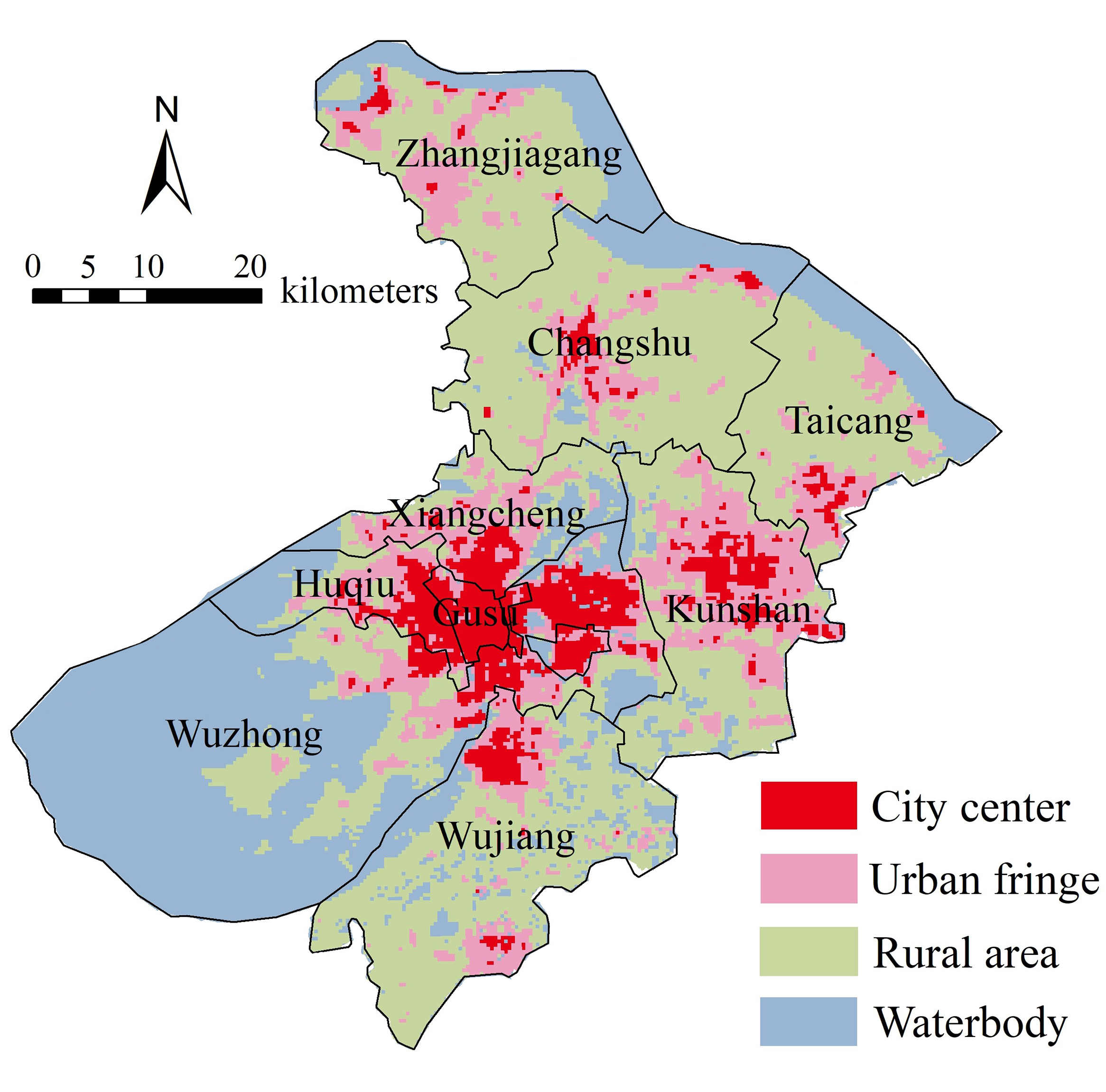

Supplement: S2 Fig — (JPG) [file pone.0332934.s002.jpg]

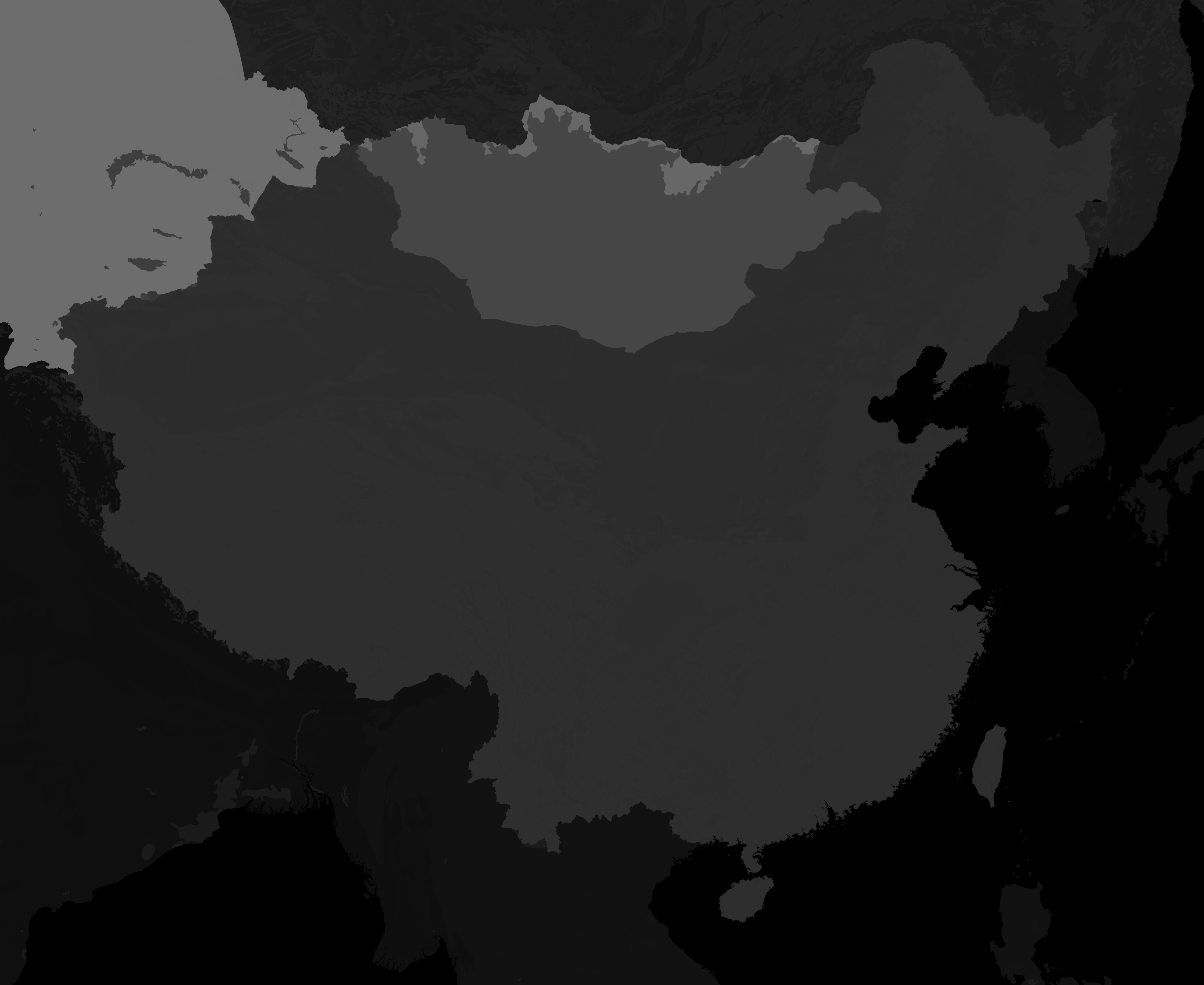

Supplement: S4 File — (ZIP) [file pone.0332934.s006.zip › S4_File.mpk/Soil/HWSD_China_Subset_v1.1/HWSD_China_Albers.img.ovr]

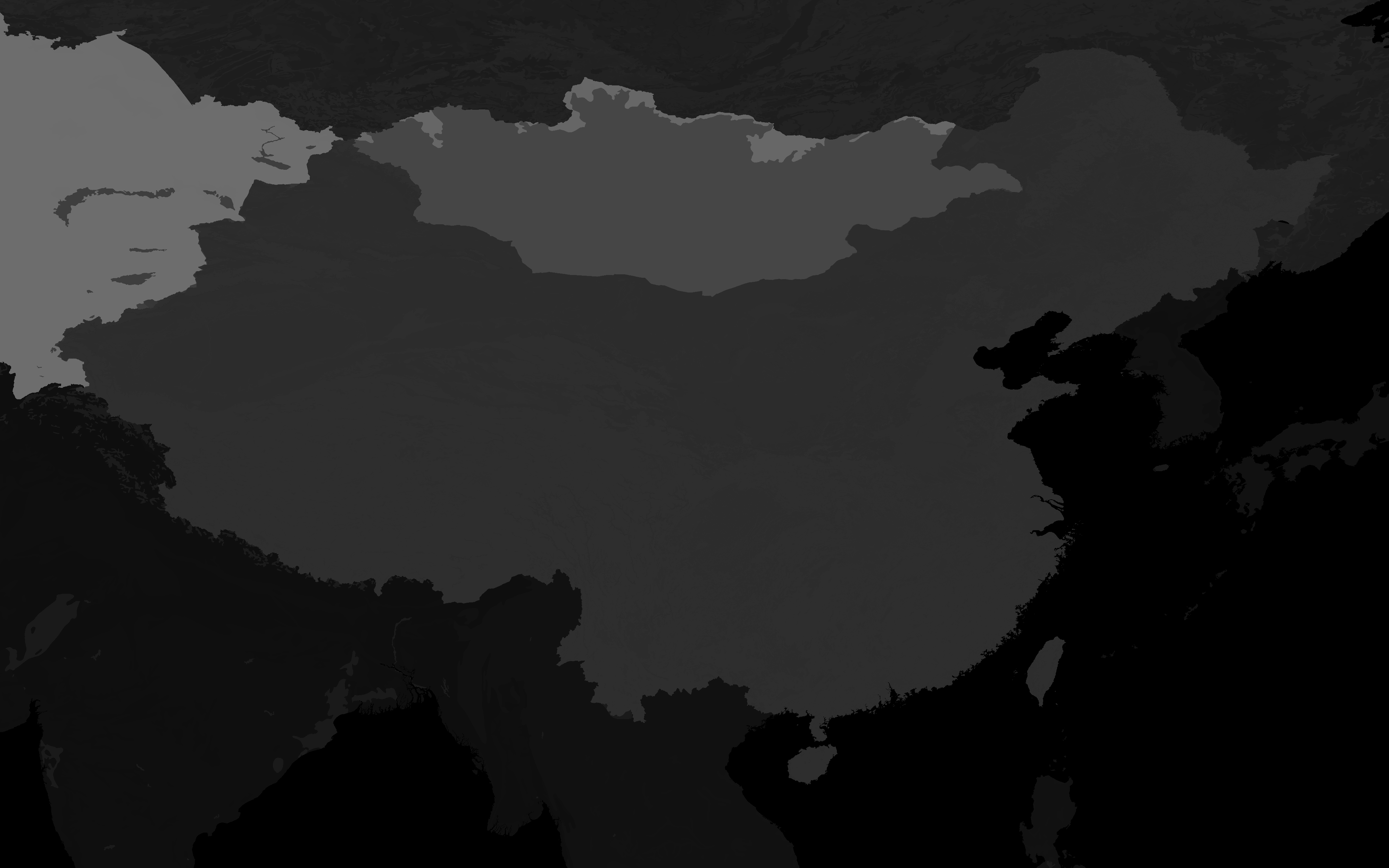

Supplement: S4 File — (ZIP) [file pone.0332934.s006.zip › S4_File.mpk/Soil/HWSD_China_Subset_v1.1/HWSD_China_Geo.img.ovr]
